# A Patient with 22q11.2 Deletion Syndrome: Case Report

**DOI:** 10.4008/jcrpe.v1i3.46

**Published:** 2009-02-06

**Authors:** Sema Kabataş Eryılmaz, Firdevs Baş, Ali Satan, Feyza Darendeliler, Rüveyde Bundak, Hülya Günöz, Nurçin Saka

**Affiliations:** 1 İstanbul University, İstanbul Faculty of Medicine, Department of Pediatrics, Pediatric Endocrinology Unit, İstanbul, Turkey; 2 İstanbul University, İstanbul Faculty of Medicine, Department of Pediatrics, İstanbul, Turkey; +90−216−331 00 21+90−216−413 61 04semakabatas@yahoo.comKanlıca Ana Çocuk Sağlığı ve Aile Planlaması Eğitim Merkezi Kanlıca İstanbul, Turkey

**Keywords:** 22q11.2 deletion syndrome, hypocalcemia, hypoparathyroidism

## Abstract

22q11 deletion is one of the most frequently encountered genetic syndromes. The phenotypic spectrum shows a wide variability. We report a boy who presented at age 11.9 years with seizures due to hypocalcemia as a result of hypoparathyroidism. FISH analysis revealed a heterozygote deletion at 22q11.2.  Positive findings for the syndrome were delayed speech development due to velofacial dysfunction, recurrent croup attacks in early childhood due to latent hypocalcemia and mild dysmorphic features. The findings of this patient indicate that 22q11 deletion syndrome may present with a wide spectrum of clinical findings and that this diagnosis needs to be considered even in patients of older ages presenting with hypocalcemia.

**Conflict of interest:**None declared.

## INTRODUCTION

22q11.2 deletion syndrome (22q11.2 DS) is one of the most frequently encountered interstitial deletion syndromes in the population. The frequency is estimated as 1/4000− 5000.(1) It is usually sporadic; however, autosomal dominant inheritance has been reported in 10−20% of the patients. The phenotypic expression shows wide variability.(2, 3) Congenital heart defect, typical facial appearance, immune deficiency due to thymic hypoplasia, palatal cleft, velofacial dysfunction, hypocalcemia associated with hypoparathyroidism, developmental and behavioral problems are the main features associated with the syndrome.

In this paper we report a patient with 22q11.2 DS who presented at age 11.9 years with hypocalcemia due to hypoparathyroidism, a condition which is unusual for this age and usually is in the differential diagnosis in early infancy.

## CASE REPORTS

An 11.9 year old male patient who had a generalized tonic seizure three days prior to presentation was seen in our unit. He was born at term by vaginal delivery, weighing 3700 g. The developmental milestones were reported to be delayed,. The patient walked at ˜2 years of age and uttered his first words at 2.5 years of age. He was offered physical therapy at that age by a pediatric neurologist. He was investigated for recurrent wheezing attacks at ˜4 years of age and was diagnosed to have gastroesophageal reflux (GER) and was treated accordingly. He underwent adenoidectomy, tonsillectomy and lacrimal canal dilatation. At 6 years of age the patient had recurrent attacks of croup and was treated with steroids. He was diagnosed to have Perthes disease at age ˜9 years. According to his family, his school performance was moderate. Family history was unremarkable.

Physical examination revealed a coarse facial appearance, with small ears, a narrow forehead, and a long face with decreased ear creases. The voice was hypernasal. Chvostek and Trousseau signs were positive. Systemic findings were otherwise normal. He weighed 53 kg (0.9 SDS), measured 142 cm (−1.2 SDS) in height and his body mass index (BMI) was 26.5 kg/m2 (4.5 SDS). He was pubertal with testicular volumes of 8 ml and pubic hair at stage 4.

Laboratory investigations presented in Table 1 revealed hypocalcemia and hyperphosphatemia. Parathyroid hormone (PTH) level was low. Thyroid hormones, cortisol and prolactin levels were normal. Other biochemical parameters, blood count and urine analysis were normal. 24 hour urine calcium was 0.42 (mg/kg/day) (normal <4 mg/kg/day).

A diagnosis of hypoparathyroidism was considered. This diagnosis, suggested by the clinical and biochemical findings, was found to be associated with 22q11.2 DS, which was shown by FISH as a heterozygote deletion. Further investigations revealed a normal echocardiography and normal B and T cell functions (not given in detail). The parents had normal FISH analysis, pointing to a sporadic mutation in our patient. Calcium replacement therapy, combined with active vitamin D was started.

**Table 1:**
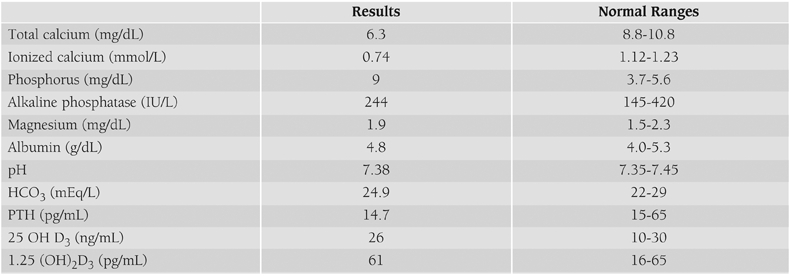
Serum biochemistry of the patient at presentation.

## DISCUSSION

Hypoparathyroidism and the resulting hypocalcemia are among the main features of 22q11.2 DS, and are observed in 70% of individuals with this deletion.(4, 5) The hypocalcemia may be of variable severity. In the severest cases the hypocalcemia is congenital. However, latent hypoparathyroidism is more frequent than persistent cases with symptomatic hypocalcemia.(6, 7) In most patients, hypocalcemia resolves around one year of age but recurs in childhood or in adolescence. In latent hypoparathyroidism PTH is secreted in sufficient quantities in basal states and serum calcium (Ca) and phosphorus (P) levels are normal. However, when Ca intake is not sufficient, especially when Ca requirements are high such as the case in infancy, adolescence or pregnancy, PTH secretion becomes inadequate and hypocalcemia becomes evident.(7, 8, 9) Recurrent croup attacks in our patient were probably related to latent hypoparathyroidism which became decompensated during puberty, due to increased calcium demand which was not met by an adequate PTH response.

Another manifestation of the syndrome in our patient was velopharyngeal dysfunction which resulted in hypernasal voice and retardation in speech development.

Congenital heart defects and immune deficiency, which are among other findings reported for patients with 22q11 DS, were not encountered in our patient. Congenital asymmetric crying facies, caused by the absence or hypoplasia of the depressor anguli oris muscle on one side of the mouth, has also been reported in 22q11 DS.(10, 11) This finding was not seen in our patient.

There is no phenotype−genotype correlation in 22q11 DS.(12, 13, 14, 15) Wide phenotypic variabilities are seen even among the family members with same mutations.(16, 17, 18) Thus early diagnosis may be difficult. A thorough clinical observation is necessary not to misdiagnose the cases with atypical findings. It is easy to make the diagnosis of 22q11 DS in the presence of congenital heart defects, palatal defect and symptomatic early onset hypocalcemia. However, in the absence of these major findings such as was the case in our patient, 22q11 DS must be considered in the differential diagnosis of developmental delay, velopharingeal dysfunction, recurrent attacks of croup and mild dysmorphic features. Early diagnosis may prompt early management of learning difficulties.

In conclusion, hypocalcemia due to latent hypoparathyroidism in late childhood and in puberty should be considered in the differential diagnosis of 22q11 DS.
